# Endogenous toxins and the coupling of gregariousness to conspicuousness in Argidae and Pergidae sawflies

**DOI:** 10.1038/s41598-018-35925-z

**Published:** 2018-12-05

**Authors:** Jean-Luc Boevé, Tommi Nyman, Akihiko Shinohara, Stefan Schmidt

**Affiliations:** 1 OD Taxonomy and Phylogeny, Royal Belgian Institute of Natural Sciences, Rue Vautier 29, B-1000 Brussels, Belgium; 2 Department of Environmental and Biological Sciences, University of Eastern Finland, P.O. Box 111, FI-80101 Joensuu, Finland; 3 Present Address: Department of Ecosystems in the Barents Region, Norwegian Institute of Bioeconomy Research, Svanhovd Research Station, NO-9925 Svanvik, Norway; 4 Department of Zoology, National Museum of Nature and Science, 4-1-1 Amakubo, Tsukuba-shi, Ibaraki 305-0005 Japan; 5 SNSB - Zoologische Staatssammlung München, Münchhausenstr. 21, 81247 Munich, Germany

## Abstract

Phytophagous insects tend to be either cryptic and solitary, or brightly colored and gregarious, as a defense against vertebrate predators. Here, we tested whether potent defensive chemicals produced *de novo* by larvae of Argidae and Pergidae sawflies have influenced the evolutionary relationship between larval appearance and levels of gregariousness. Phylogeny-based correlation analyses indicated only a weak trend for solitary species to be cryptic, and for gregarious ones to be conspicuous. Numerous Argidae were cryptic–solitary or conspicuous–gregarious, whereas most Pergidae were conspicuous–gregarious. Both families also included not truly gregarious but aggregated species, i.e. with individuals more evenly distributed on the host plant. By considering two specific morphological traits, predominant body coloration and contrasting spots on body, each one was (weakly) associated with appearance but none with gregariousness, which reflects the functional relevance of appearance as a whole. Furthermore, Argidae can display alternate appearances during successive larval instars. Finally, an independent contrasts test showed no obvious correlation between two major toxic peptides. Our results point towards diversely combined patterns of linked ecological traits in these insects. By assuming that warning coloration is more warranted against vertebrate than invertebrate predators, we suggest that the occurrence itself of toxins allowed this diversity via differing predator guilds and environmental factors, to which these insects were confronted during evolution.

## Introduction

Most insects feed on plants and are specialists^1^. They are also frequently defended against attacking predators by containing defensive chemicals, i.e. allomones^2,3^. Their capability to metabolize, store, and emit such compounds is directly linked to ecological, behavioral, morphological, and physiological adaptations^4–6^, the whole constituting a chemical defense strategy. Additionally, plants also contain deterrents and toxins, against the attack of phytophagous insects^7,8^. Thus, plant chemistry is potentially shaping the diet breadth and modulating the allomonal profile of phytophagous insects.

The biosynthetic origin of allomones should influence how ecological and defensive traits are articulated among each other. Insects rely for anti-predator defense on exogenous (i.e. sequestered) and/or endogenous (*de novo*-produced) compounds; exogenous ones are typically plant-derived and endogenous ones can be produced by endosymbiotic organisms^9–11^. At the level of plant–insect relationships, host shifts are expected to occur more frequently in insects with endogenous allomones, although a specialist species defended by plant-derived allomones can also shift from one host to another^12–15^. At the level of predator–prey interactions, the autonomy procured by endogenous allomones may impact life history traits related to anti-predator defensive strategies, but this assumption remains to be tested at a large phylogenetic scale.

For insects defended by endogenous chemicals, we can expect an increased likelihood not only of host shifts but also ‘defense shifts’, that is, switches between crypsis and conspicuousness, and across various levels of gregariousness. In insects, the chemical adaptation of unpalatability preceded the morphological adaptation of warning coloration as well as the behavioral adaptations of gregariousness, maternal care, and subsociality^16–19^. Most studies on the evolution of predator–prey interactions tend however to focus on the signaling of defenses towards birds^20^, although unpalatability itself is most probably driven by both vertebrate and invertebrate predators, as shown in leaf beetles^21,22^, tiger moths^23^, and sawflies^24^. In tritrophic approaches of ecosystems, birds preying on herbivores have been shown to top-down regulate the plant level^25^, and predatory insects, especially ants, act on plants in a same way^26–28^. This emphasizes indirectly that guilds of predators exert a high selective pressure, as plants do, on phytophagous insects.

The larvae of sawflies (Hymenoptera, Symphyta) offer an ideal opportunity to estimate the evolutionary mode of inherently intricate insect–plant and predator–prey interactions. Sawfly larvae generally feed on plant foliage whereas adults scarcely feed (e.g. on nectar). The three largest sawfly families are the Tenthredinidae with c. 5,700 species worldwide, the Argidae comprising 920 species, and the Pergidae 440 species^29,30^. In the Tenthredinidae, major defensive strategies are easy bleeding of deterrent hemolymph^31,32^ and emission of volatiles by eversible ventral glands^33^, but the group shows an overall high variety in life history traits related to defense^24^.

Here, we tested whether there is an evolutionary relationship between chemical protection, larval appearance and levels of gregariousness. To test our hypothesis, we used larvae in the sawfly families Argidae and Pergidae. The larvae of most analyzed species of these closely related groups^34^ contain hepta- and octapeptides^35,36^. These chemicals are unique among insects and, seemingly even in nature, because of a high proportion of D to L amino acids and due to the presence of phosphoserine^35^. They are lethal to vertebrates by hepatotoxicity^37–39^ and probably have deleterious effects also on invertebrates^40^. Their biosynthetic origin remains uncertain; some publications evoke the possible role of endosymbionts and at least exclude the host plant as a source of the peptides^35^. The exceptionality of the chemical structures also supports this conclusion. Thus, there are good evidences that the larvae basically rely on these endogenous toxins for defense.

Larvae of nearly all Argidae and Pergidae species feed on angiosperms, but some species rely on fungi, dead leaves, or aquatic ferns^41–44^. Both families are predominantly distributed in Asia, Australia, Africa, and the Neotropics^29^, and the hosts and habits remain unknown for many species. The general occurrence of endogenous allomones across these two related and relatively large lineages allowed studying the evolutionary consequences of the particular and potent defensive compounds on life history traits of these sawflies. The larvae are either cryptic or brightly colored, and they are distributed on their host plant by being solitary, aggregated (i.e., several individuals settled on one plant but not forming a dense group), or truly gregarious (i.e., forming a dense group)^44,45^.

Here we first infer the molecular phylogeny of Argidae and Pergidae based on DNA sequence data from two mitochondrial genes and a nuclear one, and then use the phylogeny to map host-plant and chemical data as well as the traits gregariousness and appearance. We assessed the effect of coding larval appearance as either cryptic versus conspicuous or based on two specific morphological traits, on the output of phylogenetic correlations. We also explore how peptide quantities are distributed across the reconstructed phylogenetic trees. The hypothesis is that, by generally occurring in these insects, the toxic peptides impacted the association between levels of gregariousness and appearance. We therefore discuss whether the commonness of the peptides influenced the evolution of predator–prey interactions.

## Results and Discussion

### Phylogenetic trees

**Figure 1:**
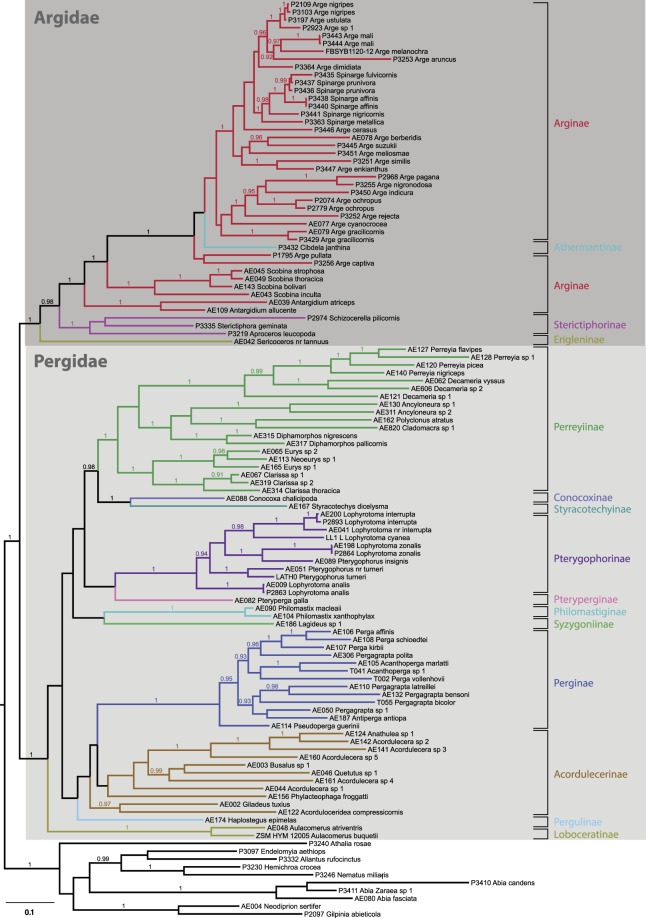
Bayesian MCC phylogeny for the Argidae and Pergidae and selected outgroup taxa representing three other sawfly families based on 1578 bp of sequence data from three genes. Subfamilies within the two ingroup families are indicated to the right of the tree, numbers above branches are posterior probabilities (only values >90% shown).

**Figure 2:**
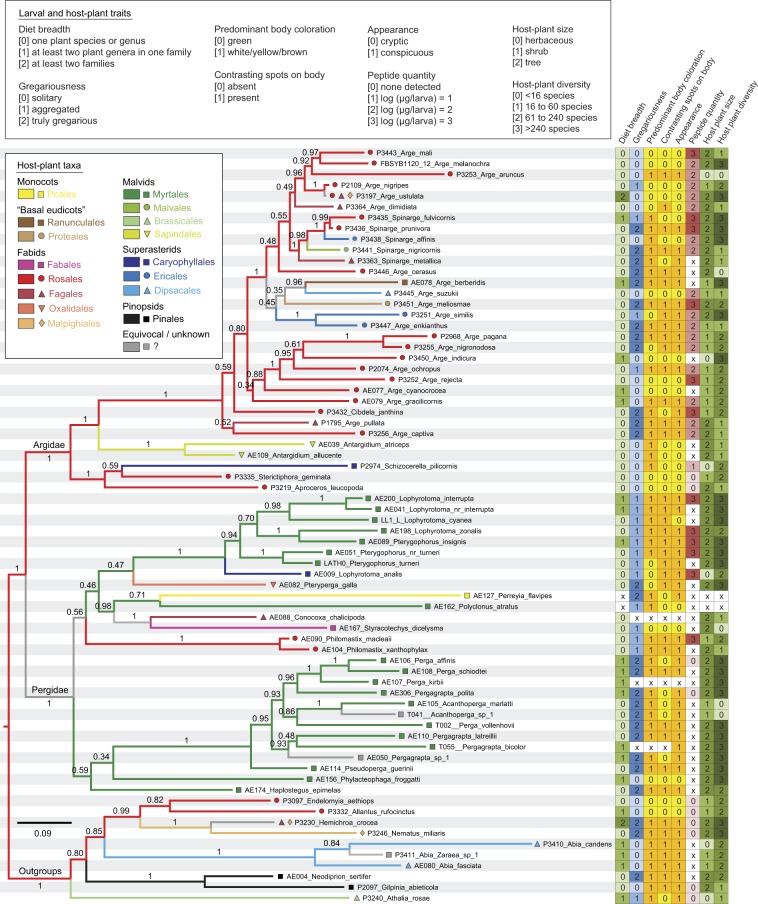
Ecological, defensive, and morphological traits of Argidae and Pergidae species in relation to their phylogeny. The tree is as in Fig. 1 but pruned to include only a single representative per exemplar species for those species that have data for at least three traits. Symbols next to tips indicate host plants of the exemplar species at the level of plant orders (see legend; classification according to^105^). Branch colors show ancestral host-use states (orders) according to maximum-parsimony optimization across the tree. Other characters and character states are indicated in the legend above the tree, and shown in the table to the right of the tree; see Supplementary Figure S1 for ML reconstructions of ancestral states in these traits.

Our Bayesian phylogenetic analysis confirms the reciprocal monophyly of the families Argidae and Pergidae (Figs 1 and 2) and strongly supports their sister-group relationship^34,46^. A previous study by Schmidt and Walter^46^ focused on the phylogeny of the Pergidae, and all of their studied taxa were used for the present analysis (Fig. 1). Major pergid subfamilies like the Pterygophorinae and Perginae came out as monophyletic, whereas other subfamilies appear to be paraphyletic, including the Perreyiinae with respect to the group that was formerly recognized as the Australian Euryinae, and, albeit with low branch support, the Acordulecerinae to the Perginae and the Australian *Phylacteophaga* (formerly Phylacteophaginae) that is nested within the Neotropical Acordulecerinae^46^. Within the Argidae, the Erigleninae genus *Sericoceros* comes out as the sister group of the remaining Argidae consisting of the Sterictiphorinae + Athermantinae + Arginae (Fig. 1). Within the latter subfamily, the tree indicates the monophyly of each of the genera *Antargidium* and *Scobina*, both being supported by high posterior probabilities. The tree also shows that *Arge* is paraphyletic with respect to the monophyletic genus *Spinarge*. By contrast, the position of *Cibdela* (Athermantinae) remains unclear because its placement within *Arge* is supported by low posterior probabilities.

### Chemical defensive strategies

Several defense mechanisms against natural enemies are known in the Argidae and Pergidae. Some of these defenses are restricted to certain species groups, such as the oral discharge of an oily fluid from a pharyngeal diverticulum in Perginae^47–49^, and volatiles emitted by non-eversible ventro-abdominal glands in species of *Arge* and *Acordulecera*^40,47^. Other mechanisms that are putatively involved in defense include the sticky secretion oozing from flask-shaped latero-abdominal glands in *Atomacera* and *Sterictiphora*^45,47^, the fluid secreted from the coxal bases in *Sericoceros*^50^, the eversible dorsal glands in *Austrosteenisia blackii*^51^, and the caudal filaments in *Lagideus podocarpus*^52^. Still other traits related to defense are known in larvae of *Schizocerella*, *Corynophilus* and *Phylacteophaga* that are leaf-miners (although *S. pilicornis* shows also an externally feeding biotype), in those of *Enjijus* that are shoot borers^44,53^, and in many Argidae larvae that raise their abdomen permanently or upon disturbance.

The most striking and regularly encountered defensive strategy among both Argidae and Pergidae larvae is to contain toxic peptides. However, these compounds were neither detected in the sterictiphorines *Sterictiphora geminata* and *Aproceros leucopoda*, nor in the pergines *Perga affinis* and *Pergagrapta polita*^36^. Pergines use mandibular structures to mechanically sequester toxic leaf compounds as a defense against natural enemies^49^.

**Figure 3:**
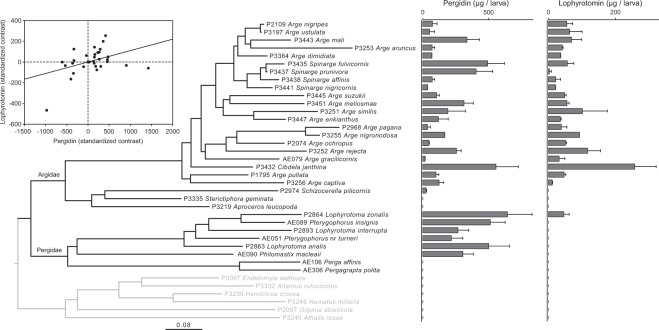
Average total amounts of pergidin and lophyrotomin in larvae of 24 Argidae, 8 Pergidae, and 6 outgroup species in relation to their phylogenetic relationships. The tree was obtained by pruning the Bayesian MCC tree to include only species from which chemical measurements were made; error bars show standard deviations of means. Standardized contrasts for the amounts of pergidin and lophyrotomin across the nodes of the phylogenetic tree are shown in the inset above the tree.

Our independent contrasts test revealed a significant cross-species correlation between the amounts of pergidin and lophyrotomin (r = 0.415, r^2^ = 0.173, *P* = 0.018; *N* = 32 ingroup species; Fig. 3). Among the species used in this test, however, *C. janthina* contains particularly high amounts of each peptide^36^, and statistical significance was lost when excluding this species from the test (r = 0.174, r^2^ = 0.030, *P* = 0.349; *N* = 31). Moreover, our stochastic mapping analyses indicated that peptide quantity was not associated with the other traits: diet breadth, gregariousness, predominant body coloration, contrasting spots on body, appearance, and host-plant diversity (Table 1). Thus, it seems that intraspecific quantities between peptides are phylogenetically unrelated, as are also peptide quantities and other defensive traits. It is surprising that peptide quantities were related to none of the three visual traits, since there are evidences that the level of toxicity is a predictor of warning coloration in ladybirds (Coccinellidae)^54^ as well as poison frogs (Dendrobatidae)^55^. Possibly this is related to the lower mobility of insect larvae compared to organisms like ladybirds and frogs. Vertebrate predators such as birds are more mobile than invertebrate predators like ants, and they may impose a higher predation pressure on mobile prey, thus linking levels of toxicity to strength of the visual signaling. However, the association between warning signal intensity and chemical defenses remains difficult to understand because it can be positive, negative, or absent depending on the studied system^56^.

**Table 1:** Overall phylogenetic correlations (*D*) between selected ecological, morphological and defensive characters, and associated uncorrected *P*-values.

Character (code)	Character (code)	*D*	*P*
Diet breadth (1)	Gregariousness (2)	0.057	0.390
Diet breadth (1)	Appearance (5)	0.036	0.832
Diet breadth (1)	Peptide quantity (6)	0.062	0.268
Diet breadth (1)	Host-plant diversity (8)	0.072	0.074
Gregariousness (2)	Predominant body coloration (3)	0.189	0.056
Gregariousness (2)	Contrasting spots on body (4)	0.156	0.058
Gregariousness (2)	Appearance (5)	0.291	**0.022**
Gregariousness (2)	Peptide quantity (6)	0.391	0.066
Gregariousness (2)	Host-plant size (7)	0.198	0.174
Predominant body coloration (3)	Contrasting spots on body (4)	0.073	0.166
Predominant body coloration (3)	Appearance (5)	0.143	**0.046**
Predominant body coloration (3)	Peptide quantity (6)	0.137	0.260
Contrasting spots on body (4)	Appearance (5)	0.142	**0.008**
Contrasting spots on body (4)	Peptide quantity (6)	0.156	0.074
Appearance (5)	Peptide quantity (6)	0.139	0.356
Appearance (5)	Host-plant size (7)	0.090	0.416
Peptide quantity (6)	Host-plant diversity (8)	0.333	0.164

The correlations were estimated by Bayesian stochastic mapping across a sample of 500 post-burnin trees pruned to include only ingroup taxa. *P*-values statistically significant at *P* < 0.05 before Holm’s sequential Bonferroni correction are given in bold. See Supplementary Table S1 for detailed results.

### Gregariousness and appearance

Among the 17 pairwise comparisons performed using stochastic mapping, only three yielded an overall statistical significance (uncorrected *P* = 0.008–0.046), but none of them remained significant after Holm’s sequential Bonferroni correction (Table 1; Supplementary Table S1). This general non-significance of the corrected results of *D* values would lead to the conclusion that none of the studied characters is associated with another. Cautiousness is even more basically requested when using methods of stochastic mapping of categorical characters, because in these methods, cases of within-clade pseudo-replication can be confounded with associations that are evolutionary independent^57^. The evolutionary association between appearance (cryptic or conspicuous) and levels of gregariousness (solitary or gregarious, respectively) is well documented in insects such as leaf beetles, lepidopterans^17,18^, and sawflies^24,58,59^. In a previous study focusing on tenthredinid sawflies, none of the phylogenetic correlations between predominant body coloration, contrasting spots on body, and other larval traits was found to be significant^24^. In our results, raw *P* values were only significant in the pairwise comparisons of appearance with gregariousness, predominant body coloration, and contrasting spots on body (Table 1); the two latter are morphological traits not always unambiguously linked to appearance^18,60^. Gregariousness was associated with none of the morphological traits (Table 1). Our results suggest that compared to appearance, the two specific morphological traits constitute a more accurate and neutral description of the visual pattern displayed by the insect, but they may be less adequate in reflecting the effective visual impact of the patterns on foraging vertebrate predators, and how this impact is amplified when the sawflies are gregarious. The statistical weakness of the gathered associations would advocate, however, for a general, alleviated explanation, that is, gregariousness and appearance evolved rather independently from each other in the Argidae and Pergidae. The mapping of traits on the phylogenetic tree (Fig. 2) revealed a discrepancy between the two families in that Argidae species are frequently cryptic–solitary or conspicuous–gregarious (i.e., aggregated or truly gregarious), whereas Pergidae are predominantly conspicuous–gregarious. The question why cryptic–solitary species are particularly rare in Pergidae remains puzzling. But one can conclude at least that the Argidae contributed more than the Pergidae in gathering the aforementioned (weakly) significant results.

Since a large majority of Argidae and Pergidae contain toxic peptides, any conspicuous species may be considered “aposematic” in the strict sense since a (chemical) defense is underlying the signaling. The next step is to understand why all the toxic species are not aposematic. Generally, insects are often preyed upon by insectivorous birds, which undoubtedly respond to visual signals and are eventually able of avoidance learning^18^. Insects are also the prey of numerous insectivorous insects for which detailed body patterns of the prey are less significant. Moreover, repellent volatiles can function as a primary defense, i.e. a signal to warn predators that rely on chemical cues during foraging and which is the case for many insects but also birds^61^. We can assume that the typical evolutionary route of a chemical defense strategy against predation by vertebrates led a chemical adaptation to be followed by warning coloration, itself preceding gregariousness. In contrast, such a route against invertebrate predators could have been shorter by ‘skipping’ the shift of appearance in that a chemical adaptation leads directly to gregariousness, if we assume, all other things being equal, that grouped individuals are better chemically defended than single ones. A predator should then face a higher total dose of distasteful compounds. In experiments not using predators but where sawfly larvae were repeatedly harassed to produce a chemical defense, however, group size decreased under high ‘attack’ intensity^62^. This supports the idea that in the evolution of insects, a conspicuous body coloration and the chemical defense itself are differently influenced by avian predators, invertebrate ones, and even more generally, by other natural enemies, host-plant quality as well as abiotic conditions^60,62^.

The assumption of a similarly important impact of vertebrate versus invertebrate predators implies the possible existence of prey insect species that are chemically defended but solitary and cryptic, or even gregarious without being necessarily conspicuous. Such species actually occur among sawflies. On the one hand, at least five Argidae species are known to contain toxic peptides while being solitary and cryptic^36^ (Fig. 2). On the other hand, gregarious and cryptic species do not occur at last larval instar, but some sawflies combine these trait states sequentially during their development, as known in a nematine and some diprionid sawflies^33,63^. However, intraspecific variation in levels of appearance is common in the Argidae, having been described in *Arge captiva*^64^, *Arge enkianthus*^65^, *Arge pagana*^40^, and *Spinarge flavicostalis*^66^. Thus, sawfly species containing toxic peptides seem to have a propensity for varying appearances during ontogeny. Although numerous Argidae and Pergidae larvae still remain to be described, the known life histories of the Argidae already include diverse combinations across and/or within species between the traits gregariousness and appearance (Fig. 2).

### Host-plant traits

The patchy distribution of an herbaceous host plant can force an insect to be aggregated rather than solitary, an explanation not holding for insects living on larger plants. Our results highlight the fact that Argidae and Pergidae are quite often aggregated although most studied species feed on shrubs or trees (Fig. 2), which suggests that aggregation is really an adapted intermediate stage of gregariousness in these sawflies. Indeed, gregariousness was uncorrelated to host-plant size (Table 1). Since endogenous chemicals may allow rather frequent host shifts^14^, these would be further enhanced if the hosts belong to a diversified group containing many taxonomically and chemically related species. However, diet breadth and host-plant diversity were uncorrelated traits (Table 1), indicating that host shifts are governed by other factors than the biosynthetic origin of allomones in Argidae and Pergidae.

### Predator–prey interactions at large paleo-geographical scale

The paradox of commonly occurring and undoubtedly toxic peptides apparently unconnected to complementary defensive traits can find some further explanations from the perspective of trends in habitat conditions and predation risks encountered by the sawflies, in geological times as well as in their recent history.

Approximately one third of the Argidae species known worldwide live in the Palearctic, whereas no single Pergidae species is recorded from this geographical region^29^. All studied Argidae species are from this region and they tend to be cryptic and solitary, or conspicuous and gregarious (i.e. aggregated or truly gregarious); by contrast, the Pergidae are rarely cryptic and solitary (Fig. 2) and we collected them mainly in Australia, although the family is also well represented in the Neotropics. *Sericoceros* is the sister-group of all other Argidae and *Aulacomerus* the one of all other Pergidae (Fig. 1). These two genera are only known from the Neotropics^29^, which may suggest that the Argidae–Pergidae split occurred in Gondwana, the Argidae subsequently extending their geographical distribution towards Laurasia (which includes the extant Palearctic). The split between the two families happened ≈ 153 million years ago, with further main splits at least in the Pergidae during the Cretaceous^46^. This is well before the adaptive radiation during the Paleocene–Eocene of ants^67^ and modern, terrestrial birds^68,69^, both groups including major taxa of predators of phytophagous insects^4^ including sawflies^24,70^. The modern birds diversified and spread in the Holarctic zone during the Paleocene or early Eocene^71^, although birds as terrestrial and arboreal insectivores are today the most diversified in tropical zones^72^. Ants are also at present especially diversified and abundant in the (sub)-tropics where they forage at all vegetation strata^73,74^, and, more generally, predation inflicted by invertebrates is pronounced at low latitudes^75^. Thus, both Argidae and Pergidae were confronted to vertebrate as well as invertebrate predators during longstanding geological times, but the Argidae fauna from the Palearctic may be comparatively more subjected to avian predation since the Paleocene–Eocene.

Open and heterogeneously structured habitats are more common in the temperate and subarctic zones of the Palearctic than in the (sub)-tropical zones of the Southern Hemisphere that are partly dominated by dense and multi-strata rain forests^76^. In larvae of the lepidopteran genus *Papilio*, the signal environment is an important determinant in the evolution of aposematic coloration, and such advertising signals are likely more efficient in open than closed environments^77^. Thus, our results may reflect a rather frequent need for Argidae, at least from the Palearctic, to be defended against avian predators and which is allowed by the chemical defense eventually leading to conspicuousness and a gregarious behavior. Comparatively, the defense strategy of the Pergidae would be less impacted by birds, the toxic peptides nevertheless allowing the sawflies to become gregarious in response to predation by invertebrates.

## Conclusions

Phylogenetic correlations potentially reveal the existence of relevant ecological and/or functional associations across traits^57^. However, a trait in a prey can variably evolve under the selective pressures of several differing guilds of predators, conversely undermining the significance of associations between traits. In the Argidae and Pergidae, none of the tests of phylogenetic correlation remained significant after statistical correction, with only uncorrected results indicating gregariousness and two specific morphological traits to be associated with appearance. The emergence of each of the traits appearance and gregariousness requires a (chemical) defense^78^, a prerequisite fulfilled in most representatives of the two families. The widespread occurrence of original and potent peptides may have allowed these sawflies to adjust the ecological traits of conspicuousness and gregariousness in response to specific ecological conditions, and more in terms of predator–prey than insect–plant relationships. Although gregariousness rarely evolves without warning coloration in a prey lineage^79^ under the impact of vertebrate predators, we suspect the likelihood of a direct causal relationship linking chemical defense to gregariousness to be higher when considering invertebrate predators. To the extent that the evolution of Argidae and Pergidae is driven by both types of predators, the combined selective pressures can explain the weak association between conspicuousness and gregariousness. Moreover, peptide quantities were related to none of the three visual traits.

Plant-derived and endogenous chemical defenses of phytophagous insects do not differ in the magnitude of their effects on predators^21^. The availability of the endogenous chemicals used by the sawflies studied here may have contributed to the geographical expansion and radiation of these insects. Yet, such conclusions are subject to caution due to incompleteness of our knowledge about the biology and ecology of the sawflies, especially for species from the Southern Hemisphere and that remain rarely collected. Nevertheless, we identified multi-guild predation as a determinant in the evolution of these sawflies, probably in combination with environmental factors that differ between habitats at a large paleo-geographical scale.

## Methods

### Taxon sampling

Our phylogenetic analysis was based on 38 Argidae and 60 Pergidae species collected mainly in Europe, Japan, Australia, and South America (Supplementary Table S2). The exemplar species represent four out of the seven subfamilies of Argidae, and ten out of the twelve subfamilies of Pergidae. Worldwide, 39% of Argidae species belong to the genus *Arge*^29^, from which 22 species were included in our analysis. As outgroups, we sampled ten species from three sawfly families (Supplementary Table S2). Vouchers are kept at the Royal Belgian Institute of Natural Sciences, and SNSB - Zoologische Staatssammlung München.

### DNA sequencing

DNA was extracted from a leg taken off specimens stored in ethanol or from dry mounted museum specimens using Qiagen DNeasy Tissue Kits according to the manufacturer’s protocol, except that lysis took several hours or overnight. Three gene regions were amplified: (1) the 5′ end of the mitochondrial cytochrome *c* oxidase 1 gene (COI; 658 bp), (2) a fragment of the mitochondrial 16 S rDNA gene (397 bp), and (3) the D2 loop of the nuclear 28 S rDNA gene (523 bp). For primers and sequencing protocols, see Schmidt and Walter^46^. Sequences were edited using Sequencher version 4.9 (Gene Codes Corp., Ann Arbor, MI, USA) and are available in GenBank (accession numbers KF318463–KF318519, KC567820–KC567882, KF445006–KF445070).

### Phylogeny reconstruction

We aligned the ribosomal sequences (28S-D2 and 16 S rDNA) using the T-Coffee software package^80^, which allows incorporating information on secondary structure predicted using RNAplfold, which is part of the ViennaRNA package^81^. COI sequences were aligned with MUSCLE^82^. Bayesian inference was conducted with MrBayes version 3.2.2^83^. The combined analyses employed separate, unlinked substitution models for each gene region, and distinct models for codon positions of the COI region. In addition, gaps were coded as binary characters using FastGap version 1.2^84^. For nucleotides, a mixed substitution model with gamma-categorized rate variation was employed, whereas gaps were included as a separate partition of variable, gamma-corrected binary characters. Two independent runs with one cold and three heated chains were run for five million generations, after which the average standard deviation of split frequencies had reached values lower than 0.01. Trees were sampled every 1,000 generations and the suitability of a relative burn-in fraction of 0.25 was examined using Tracer 1.6, prior to calculating a Bayesian consensus tree including all compatible groups.

### Character coding and chemical measurements

To infer the evolutionary history of ecological and defensive traits within Argidae and Pergidae, we gathered data on larval traits and host features for the species included in the phylogenetic tree. The data were categorized and coded into two to four states for each character (see Fig. 2).

To determine diet breadths, species-specific host plants were compiled from reliable literature sources (e.g.^30,85,86^). Data on larval gregariousness and appearance were extracted from standard works on sawflies^30,45^, specific works on Argidae (e.g.^85^, and literature therein), and unpublished observations and sources. Since gregariousness as well as appearance can vary from one larval instar to another (e.g.^40,85^), only the last instar preceding the eonymph stage was considered. Gregariousness was coded into three states: solitary, aggregated (i.e., larvae distributed on a plant, generally less than three per leaf), or truly gregarious (larvae on one leaf or several adjacent leaves).

In a previous phylogenetic analysis of tenthredinid sawflies^24^, larval appearance was indirectly studied via two morphological traits, predominant body coloration and the presence of contrasting spots on body. The predominant body coloration corresponded to the major color of the body, here being either green, white, yellow, or brown. Since the larvae feed on the foliage, green was set as one state, the three other body colors as another state because all three appear in contrast to the close environment. Spots on the body were considered in relation to their contrasting effect with the predominant body coloration; such spots were generally dark but could be bright and whitish. These two morphological traits can be coded more objectively than appearance *per se*, because determining whether a species is cryptic or conspicuous partly depends on factors such as the immediate visual environment (background) of larvae, larval body size, visual capabilities of the observer, etc.^54,77,87–91^. To assess the impact of the coding method on the results, we included here the two aforementioned morphological traits plus the more subjective one, appearance, as either cryptic or conspicuous. We judged the appearance on the basis of the general visual effect rendered by the larva settled on the foliage. Note that aposematism in the strict sense is linked to an underlying defense^4^, which was an attribute not considered when determining the appearance. Furthermore, the link between the two morphological traits and appearance is not always obvious. For instance, instead of rendering the prey more conspicuous, dark body marks can actually enhance crypsis, and their presence or absence can be related to other functions than defense^18,60^.

Besides the traits directly linked to the sawfly larvae, we included in our analyses two specific plant traits that may however influence larval defensive strategies. Data about host-plant size and diversity were obtained from standard floras (e.g.^92^). Host-plant diversity refers here to the maximum number of known (extant) species of a plant genus. If a sawfly species feeds on more than one plant genus, this number was then the sum of all the species in the corresponding plant genera.

Data on peptide quantities (in µg per larva) were available for 28 Argidae and eight Pergidae species^36^, whereas earlier literature sources^35,93^ unambiguously identified and quantified the peptides in only one argid and three pergid species. Cumulative quantities of the three major peptides, pergidin, 4-valinepergidin and lophyrotomin, were coded. No complete information exists about possibly differing toxicities of these peptides on a predator, except about the lethal dose (LD) for mice by intra-peritoneal injection: LD_50_ ≈ 10 mg pergidin/kg, and 2 mg lophyrotomin/kg^35,94^. Such toxicity tests clearly do not reflect natural predatory-prey interactions^95^. We cannot exclude that the two compounds strongly differ in their feeding deterrence and/or toxicity, knowing however that these hepta- and octapeptides are closely related^35,36^. We assumed therefore that the chemical defense efficiency of a species, all other things being equal, is proportional to the total amount of peptides it contains.

### Ancestral-state reconstructions and phylogenetic correlation analyses

In our ancestral-state reconstructions, we took phylogenetic uncertainty into account by maximum-likelihood (ML) optimizing states of the focal ecological, morphological, and chemical characters across 500 trees sampled from the Bayesian posterior distribution. These trees were obtained by randomly subsampling 250 post-burnin trees from each of the two MrBayes runs using the sample.trees function of the package ape^96^ in R^97^. The ape package was also used for pruning the trees to include a single representative of each species for which states were known for at least three characters. ML optimization was done using Mesquite v. 3.40^98^, and the results were summarized on the nodes of the similarly pruned Bayesian maximum clade credibility (MCC) tree. Because some of the most polyphagous exemplar species use several plant orders and multistate taxa cannot be ML optimized in Mesquite, ancestral host use was inferred by simple maximum-parsimony optimization of plant orders across nodes of the MCC tree.

Correlations between characters and character states were inferred by Bayesian stochastic mapping^99,100^ in SIMMAP v. 1.5.2^101^. Only ingroup species were included in these analyses and, in order to accommodate phylogenetic uncertainty, all correlation analyses were performed across 500 pruned post-burnin trees (rescaled to a length of 1). Seventeen character-pair comparisons were selected out of the 28 possible ones among the eight focal traits (Supplementary Table S1; Table 1). This selection was guided by supposing an evolutionary: (*i*) independence between the occurrence of toxic peptides and specific visual traits (gregariousness and appearance), (*ii*) non-independence between appearance and morphological traits (predominant body coloration and contrasting spots on body), and (*iii*) independence between plant- and sawfly-related features. State-by-state associations between characters were evaluated based on the *d*_ij_ statistic, which measures co-occurrence of states i and j across branches in relation to the expectation under independent evolution^100^, and overall character correlations were measured using statistic *D*, which is the sum of the absolute values of individual *d*_ij_’s between characters^100^. All characters were treated as unordered, and pairwise correlation analyses were configured using bias and rate priors determined using the MCMC-based approach of Schultz and Churchill^102^ in SIMMAP. For binary characters, best-fitting priors for the bias parameter (=beta distribution parameter α) and the overall evolutionary rate parameters (=gamma distribution parameters α and β), were determined based on the pruned MCC tree, which had been rescaled to a length of 1. MCMC runs were performed using the default number of distribution discretization categories (31 for the bias parameter and 60 for the rate parameters), cycles (100,000), sampling frequency (200), burnin (10,000), and upper rate bound (1,000). Results were extracted in R v. 3.3.2^97^ using the sumprmcmc.r script provided in the SIMMAP installation package. In the case of multistate characters, we used an empirical prior for the bias parameter, while rate-parameter priors were determined as described for binary characters. The number of samples, prior draws, and predictive samples were set to 1, meaning that both observed and predictive sample sizes equaled 500 for each character pair.

### Independent contrasts test

The major toxic peptides are pergidin and lophyrotomin in Argidae, whereas pergidin and 4-valinepergidin in Pergidae^36^. Such data about peptide contents were gathered using the last larval instar, and are expressed in absolute amounts of µg per individual. In the context of predator–prey interactions, an absolute amount of peptides well reflects the actual (maximum) amount to which a predator is confronted while attacking a prey item. The amount is potentially determined by factors of the sawfly, such as species, body size, population, etc.^35,36^. To analyze the distribution of defensive chemicals in relation to the phylogenetic position of species, Felsenstein’s^103^ independent contrasts method was applied on the published dataset^36^ of peptide quantities, using specifically the quantities of pergidin versus lophyrotomin. The peptide 4-valinepergidin was discarded due to the higher number of both chemical and genetic data available for Argidae (24 species) than for Pergidae (8). The tree used in these analyses was obtained by pruning the Bayesian MCC tree to include only species for which chemical data were available, and contrasts were calculated using the PDAP:PDTREE package v. 1.15^104^ in Mesquite.

## Electronic supplementary material

Figure S1

Table S1

Table S2

## Data Availability

The datasets generated during and/or analyzed during the current study are available from the corresponding author on request.
