# Supplementary material for: Endogenous toxins and the coupling of gregariousness to conspicuousness in Argidae and Pergidae sawflies
Source: Sci Rep. 2018 Dec 5;8:17636. doi: 10.1038/s41598-018-35925-z (PMC6281571; doi:10.1038/s41598-018-35925-z)

Endogenous toxins and the coupling of gregariousness to conspicuousness in Argidae and Pergidae sawflies

Jean-Luc Boevé, Tommi Nyman, Akihiko Shinohara, Stefan Schmidt

Reconstruction of ancestral states in various ecological, morphological, and chemical characters of Argidae and Pergidae larvae, estimated using maximum-likelihood optimization of states across 500 trees sampled from the Bayesian posterior distribution: (A) diet breadth; (B) gregariousness; (C) predominant body coloration; (D) contrasting spots on body; (E) appearance; (F) peptide quantity; (G) host-plant size; and (H) host-plant diversity.

(A) Diet breadth

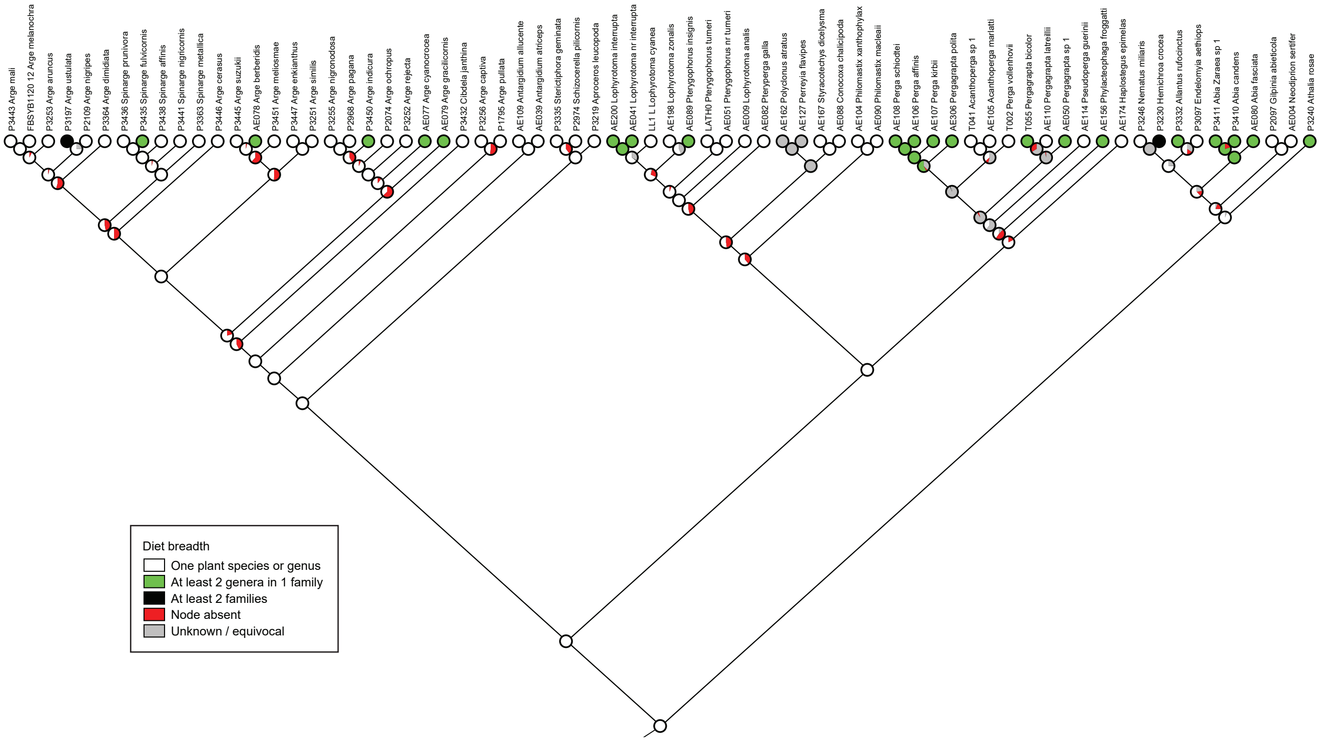

(B) Gregariousness

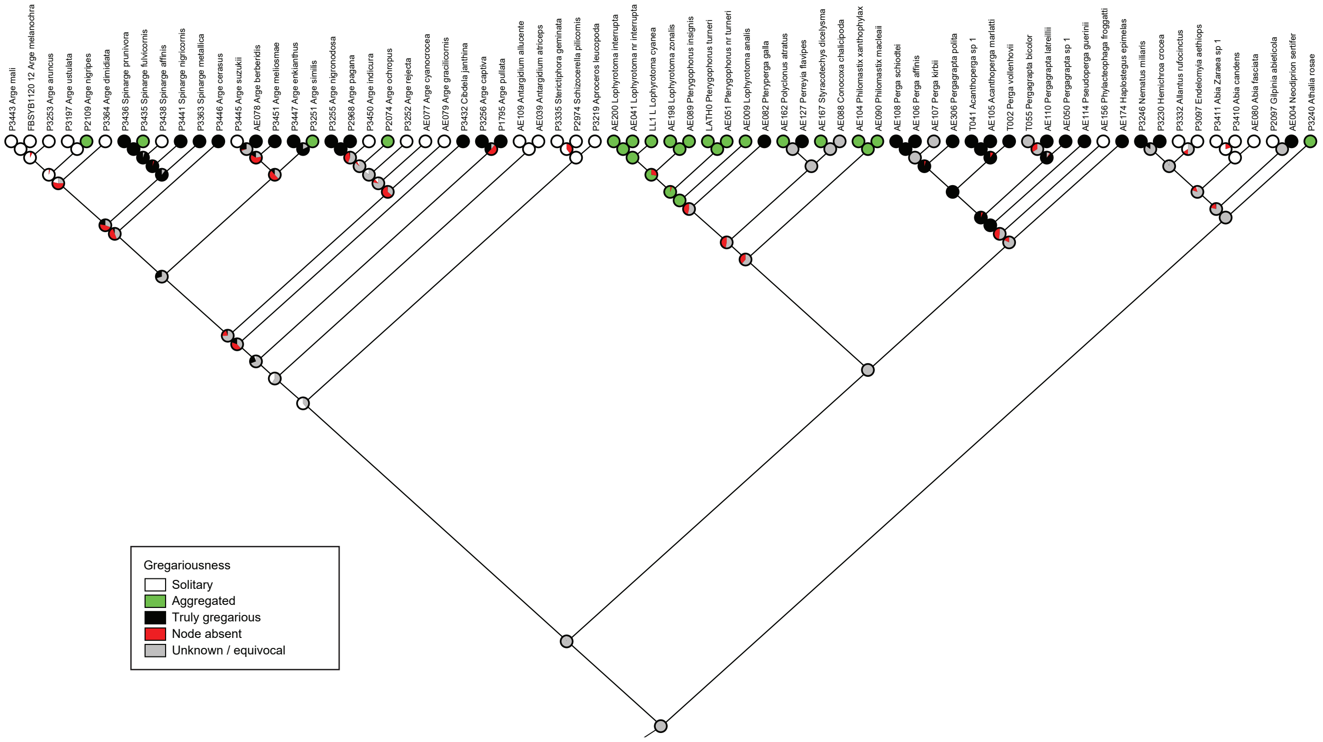

(C) Predominant body coloration

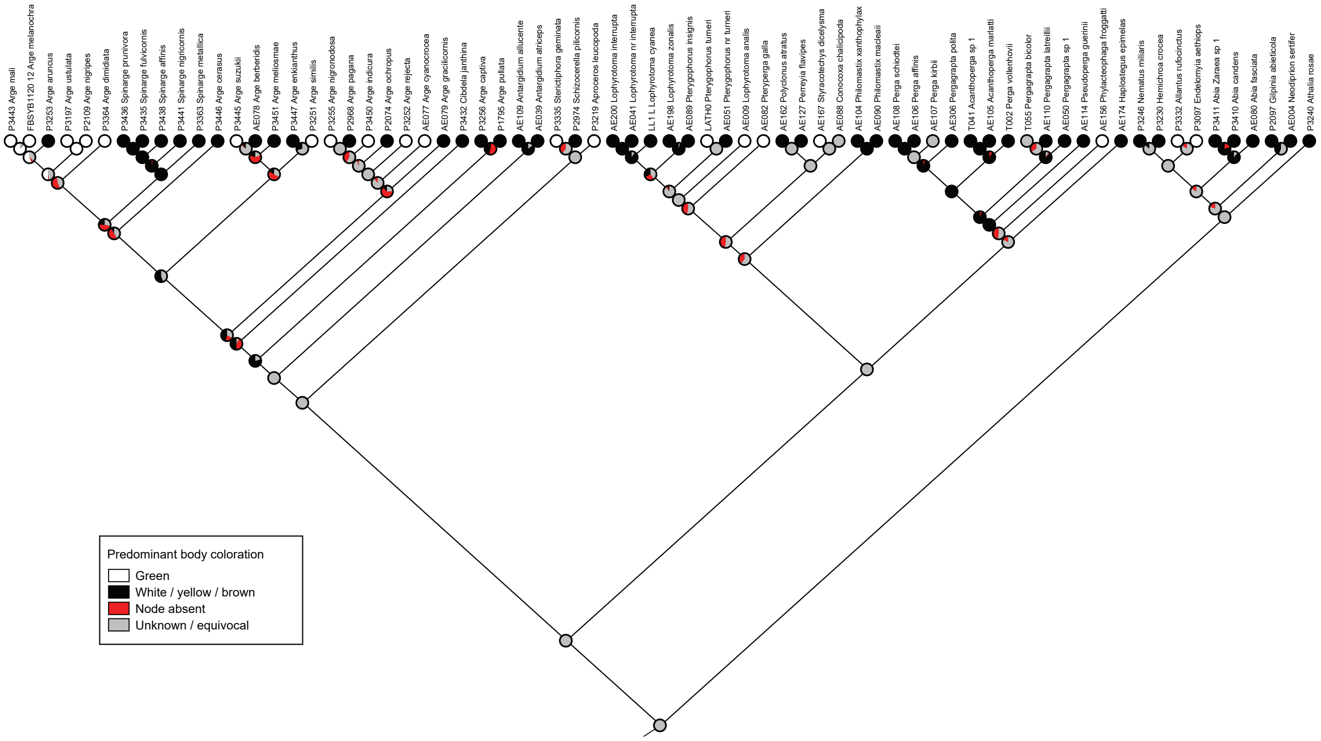

(D) Contrasting spots on body

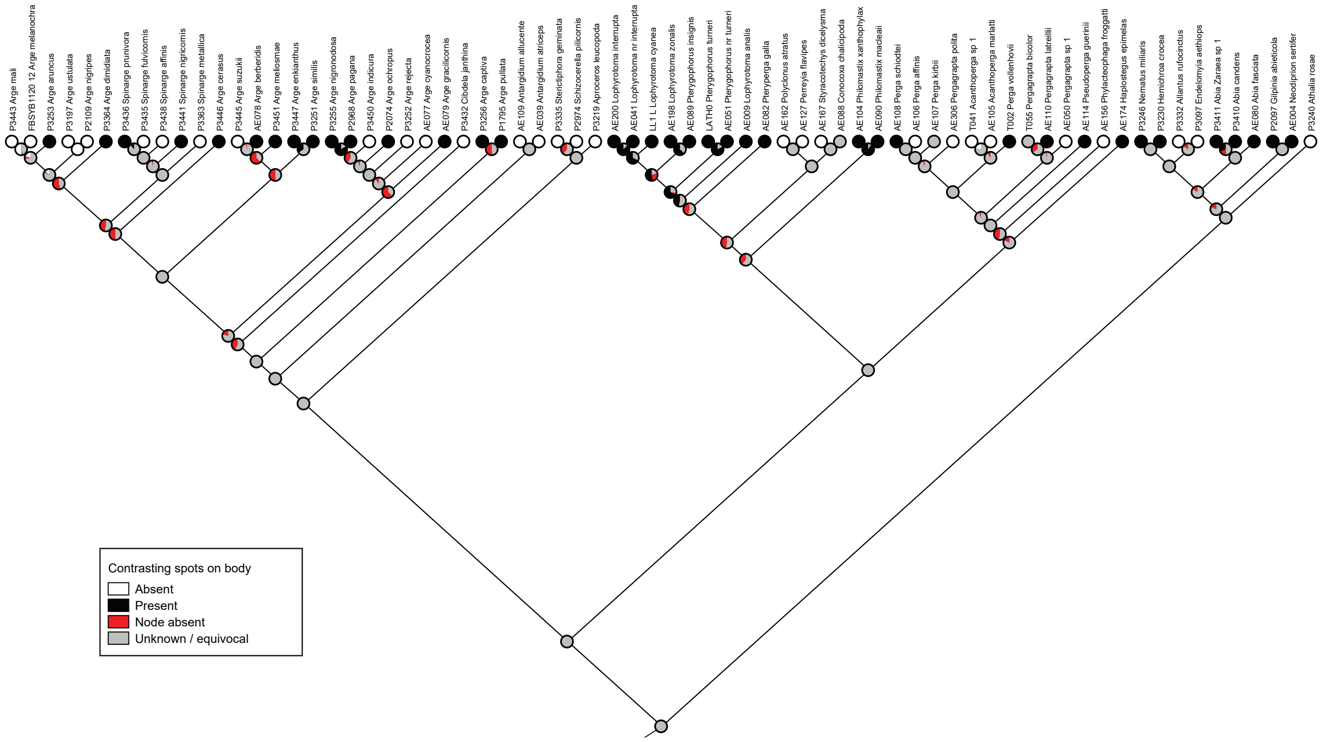

(E) Appearance

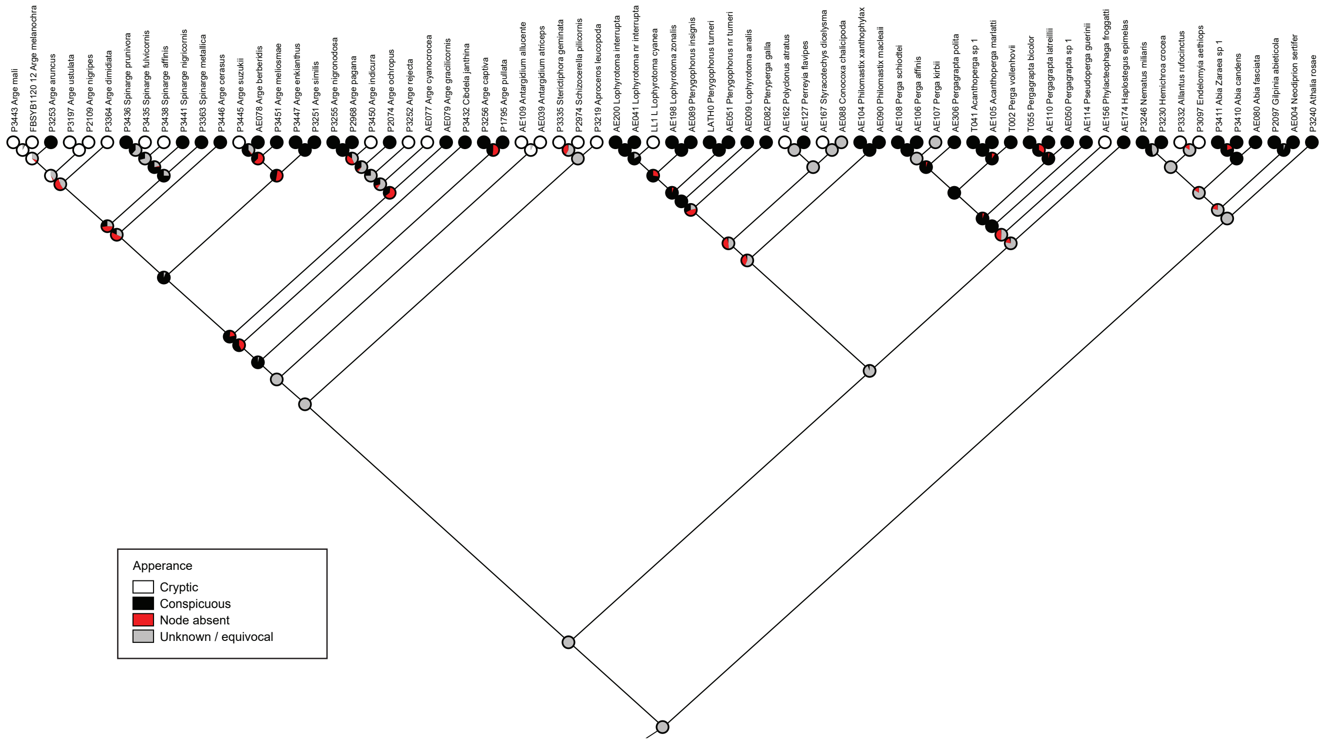

(F) Peptide quantity

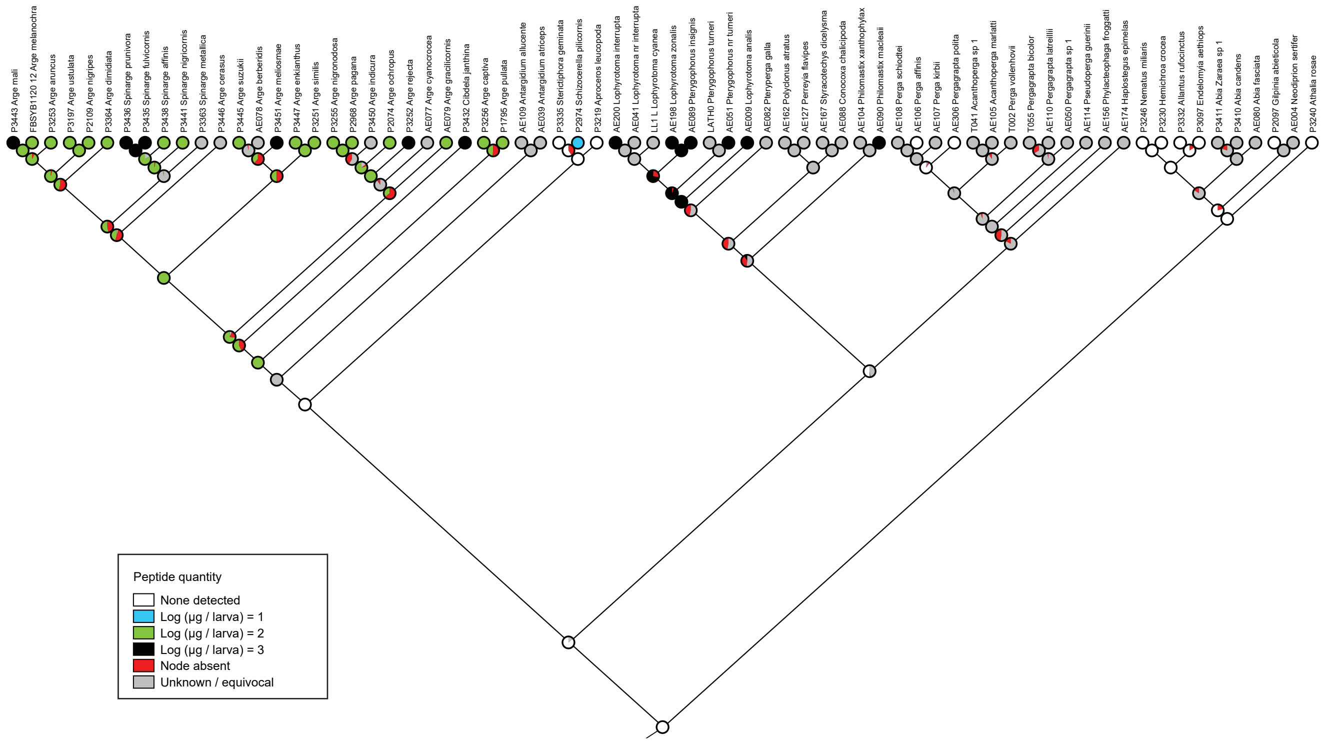

(G) Host-plant size

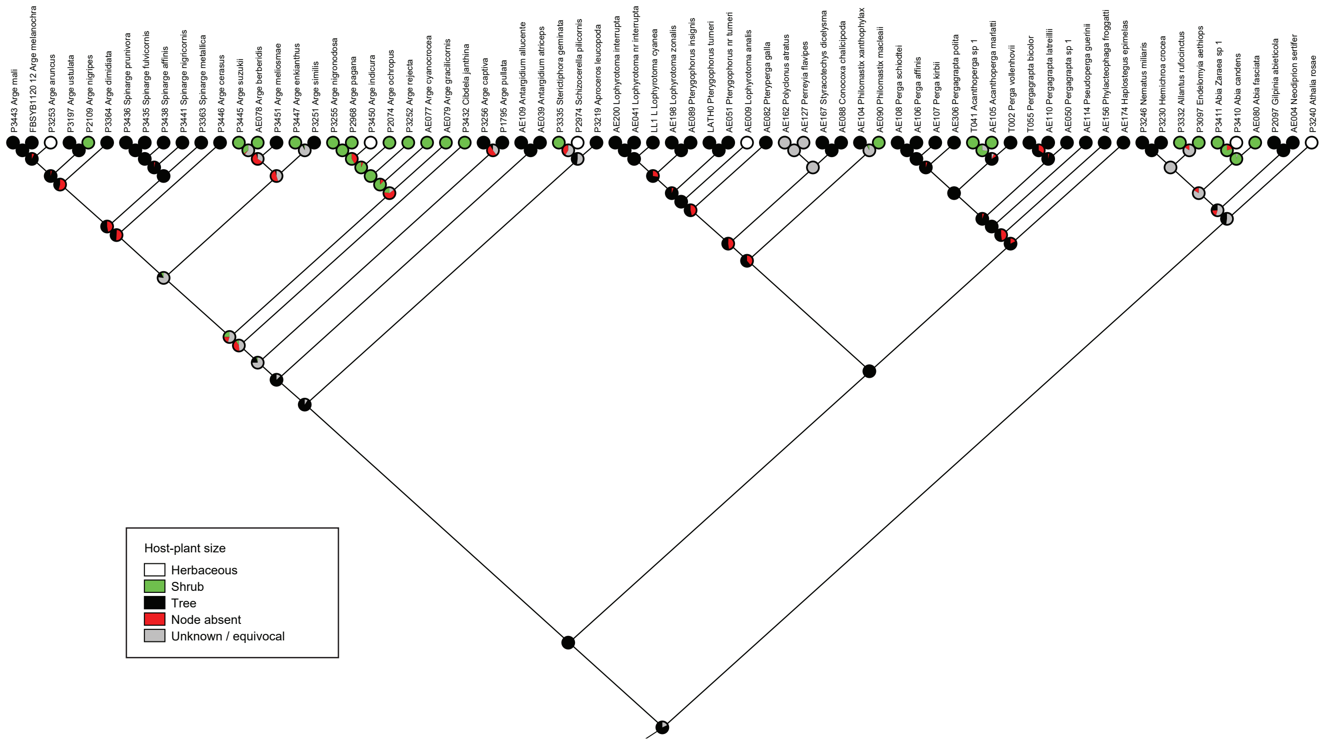

(H) Host-plant diversity

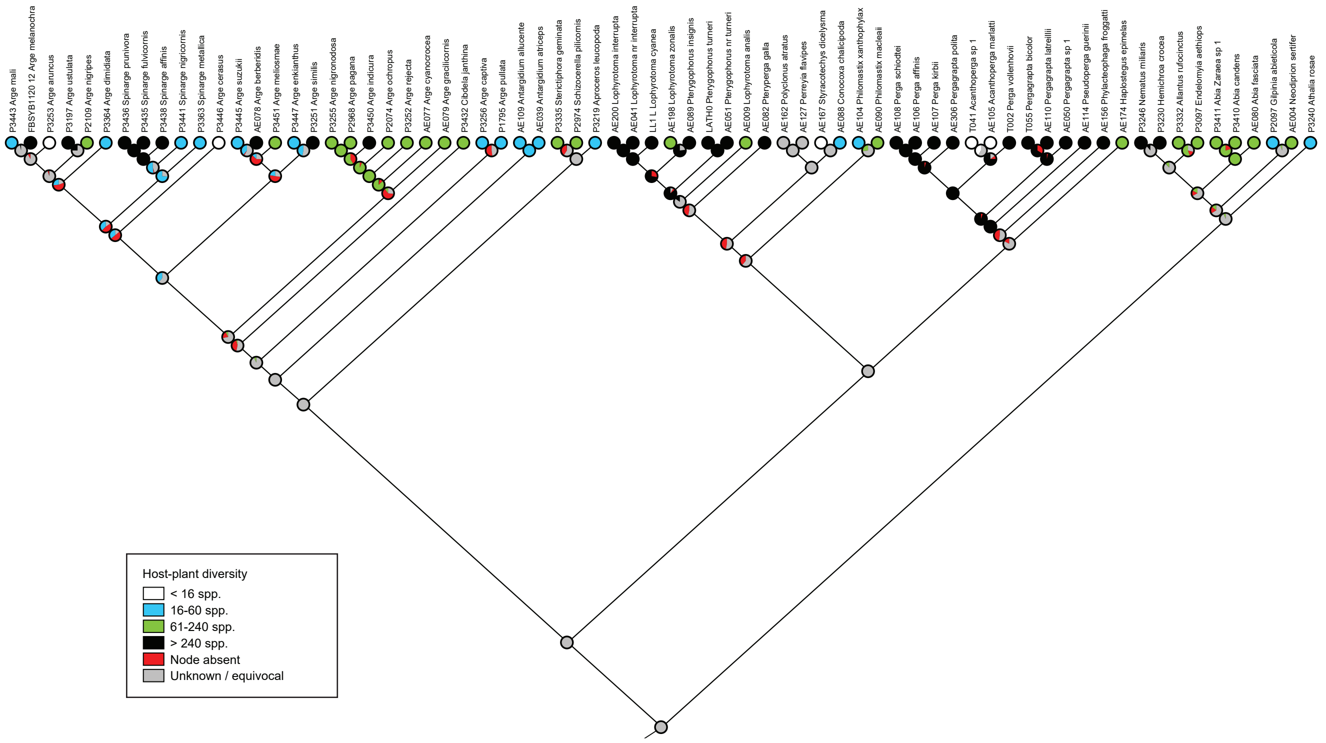

Supplement: Supplementary file 1 — Figure S1 [file 41598_2018_35925_MOESM1_ESM.pdf]
